# ADP-Ribosylation Regulates the Signaling Function of IFN-γ

**DOI:** 10.3389/fimmu.2021.642545

**Published:** 2021-03-08

**Authors:** Stephan Menzel, Tomas Koudelka, Björn Rissiek, Friedrich Haag, Catherine Meyer-Schwesinger, Andreas Tholey, Friedrich Koch-Nolte

**Affiliations:** ^1^Institute of Immunology, University Medical Center Hamburg-Eppendorf, Hamburg, Germany; ^2^Institute of Experimental Medicine, AG Systematic Proteome Research and Bioanalytics, Christian-Albrechts-Universität, Kiel, Germany; ^3^Department of Neurology, University Medical Center Hamburg-Eppendorf, Hamburg, Germany; ^4^Institute of Cellular and Integrative Physiology, University Medical Center Hamburg-Eppendorf, Hamburg, Germany

**Keywords:** purinergic signaling, ADP-ribosylation, T cells, interferon-gamma, NAD^+^

## Abstract

Murine T cells express the GPI-anchored ADP-ribosyltransferase 2.2 (ARTC2.2) on the cell surface. In response to T cell activation or extracellular NAD^+^ or ATP-mediated gating of the P2X7 ion channel ARTC2.2 is shed from the cell surface as a soluble enzyme. Shedding alters the target specificity of ARTC2.2 from cell surface proteins to secreted proteins. Here we demonstrate that shed ARTC2.2 potently ADP-ribosylates IFN-γ in addition to other cytokines. Using mass spectrometry, we identify arginine 128 as the target site of ADP-ribosylation. This residue has been implicated to play a key role in binding of IFN-γ to the interferon receptor 1 (IFNR1). Indeed, binding of IFN-γ to IFNR1 blocks ADP-ribosylation of IFN-γ. Moreover, ADP-ribosylation of IFN-γ inhibits the capacity of IFN-γ to induce STAT1 phosphorylation in macrophages and upregulation of the proteasomal subunit ß5i and the proteasomal activator PA28-α in podocytes. Our results show that ADP-ribosylation inhibits the signaling functions of IFN-γ and point to a new regulatory mechanism for controlling signaling by IFN-γ.

## Highlights

- Shed ARTC2.2 ADP-ribosylates several cytokines with a preference for IFN-γ.
- ADP-ribosylation of IFN-γ at Arg128 inhibits its function.
- Our study uncovers a novel pathway to regulate IFN-γ signaling by ADP-ribosylation.

## Introduction

ARTC2.2 is a toxin-related, GPI-anchored ecto-ADP-ribosyltransferase expressed on the surface of murine T cells (1, 2). ARTC2.2 catalyzes arginine-specific ADP-ribosylation of P2X7 and other cell surface proteins in response to NAD^+^ released from damaged cells (3, 4). Akin to phosphorylation, ADP-ribosylation is a reversible posttranslational modification that regulates the function of target proteins (5, 6). The targets of ADP-ribosylation are mainly ecto-domains of membrane proteins like P2X7, integrins or Fc-gamma-receptors (4, 7). Further, ADP-ribosylation of soluble proteins has been described previously, for example, ARTC1-mediated ADP-ribosylation of HNP-1 reduces the antimicrobial activity of HNP-1 (8). We previously observed that ARTC2.2 is shed by a metalloprotease following T cell activation as well as upon activation of the P2X7 ion channel (9, 10). Release of ARTC2.2 from the cell surface redirects the enzymatic activity of ARTC2.2 from cell surface bound membrane proteins to soluble proteins in the extracellular milieu (10). We hypothesized that the release of ARTC2.2 from the cell surface into the inflammatory environment has immunoregulatory functions by regulating cytokine signaling. ARTC2.2 is expressed in mice and other rodents. In humans and other primates ARTC2.2 represents a nonfunctional pseudogene (11) but other members of the ADP-ribosytransferase family, for example, ARTC1 are expressed (12).

The goal of this study was to identify potential target cytokines of shed ARTC2.2. Using radio-ADP-ribosylation and mass spectrometry analyses we identified IFN-γ as a major target of shed ARTC2.2. We identified the ADP-ribosylation site on IFN-γ and assessed the effects of ADP-ribosylation on the signaling functions of IFN-γ by flow cytometry and immunoblot analyses. Our results show that ADP-ribosylation inhibits the signaling functions of IFN-γ and point to a new regulatory mechanism for controlling signaling by IFN-γ.

## Materials and Methods

*Mice and cells* – C57BL/6 mice were bred at the animal facility of the University Medical Center (UKE). ARTC2^−/−^ mice (13) were backcrossed to C57BL/6 wild-type (WT) mice for more than 12 generations. All animal experiments were performed in accordance with local regulations (registration number ORG153).

DC27.10 lymphoma cells (14), kindly provided by Bernhard Fleischer, Bernhard Nocht Institute for Tropical Medicine, Hamburg, were cultured in RPMI-1640 supplemented with 10% FCS, 2 mM glutamine, 2 mM sodium pyruvate. An expression construct encoding ARTC2.2 (Genebank AJ489297) with an N-terminal FLAG-tag was transfected into DC27.10 lymphoma cells using jetPEI transfection reagent (Q-Biogen) and stable transfectants were obtained by selection of neomycin-resistant cells and cell sorting (4).

*Radio-ADP-ribosylation assays* – Recombinant cytokines were obtained from Immunotools, recombinant IFNR1-Fc was obtained from R&D Systems. Recombinant murine cytokines (0.1–0.5 μg) and serum proteins from ARTC2−/− mice (50 μg) were incubated with 20 ng shed ARTC2.2 and 1 μM 32P-NAD^+^ for 20 min. Reactions were stopped by addition of NuPAGE SDS sample buffer and SDS-PAGE was performed using Novex NuPAGE precast 12% Bis-Tris gels (Invitrogen). Gels were stained with Coomassie brilliant blue and dried in cellophane sheets. Dried gels were subjected to autoradiography by exposure of an X-ray film (Hyperfilm, GE-Healthcare) at −80°C.

*Purification of ARTC2.2* – Shed ARTC2.2 was purified as described before (10). Briefly, DC27.10 cells stably transfected with ARTC2.2 were treated for 20 min at 37°C with 1 mM ATP. ARTC2.2 was purified from the cell supernatant by affinity chromatography on immobilized anti-FLAG mAb M2 (Sigma) and concentrated via ultra centrifugal filters (Amicon).

*Mass spectrometry analyses* – Recombinant IFN-γ (1 μg) was incubated with 5 μM NAD^+^ in the absence or presence of purified shed ARTC2.2 (50 ng) for 60 min at 37°C. After SDS-PAGE, the excised IFN-γ bands were digested with either chymotrypsin or endoproteinase Glu-C (Glu-C). Resulting peptides were separated and analyzed by nano-ion pair reversed phase HPLC coupled online to an Orbitrap LTQ Velos mass spectrometer. Collision-induced dissociation (CID), Higher-energy collisional dissociation (HCD) and MS^3^ experiments were used to identify ADP-ribosylation sites.

*IFN-*γ *signaling assays –* Recombinant ADP-ribosylarginine hydrolase ARH1 was produced as described previously (15). Murine IFN-γ (1 μg) was ADP-ribosylated by incubation for 60 min at 37°C with 10 μM NAD^+^ and ARTC2.2 (50 ng). For de-ADP-ribosylation, ADPR-IFN-γ was further incubated with an excess of ARH1 (1 μg) for 60 min at 37°C. For monitoring IFN-γ induced phosphorylation of STAT1 in peritoneal macrophages, C57BL/6 mice were sacrificed and peritoneal macrophages were harvested *via* peritoneal lavage. 2 × 10^5^ cells were incubated at 37°C in RPMI containing serial dilutions of IFN-γ, ADPR-IFN-γ or de-ADP-ribosylated IFN-γ (de-ADPR-IFN-γ). After 30 min, cells were washed, fixed with 2% PFA and stained with FITC-conjugated anti-CD11b (mAb M1/70, Biolegend). Cells were then washed, permeabilized at 4°C using Phosflow Perm Buffer III (BD) and stained with anti-phospho STAT1 (mAb pY701, BD PhosFlow) on ice. Cells were washed twice and analyzed by flow cytometry.

For monitoring IFN-γ induced upregulation of immunoproteasome components, terminally differentiated mouse podocytes (16) were incubated for 24 h with IFN-γ, ADPR-IFN-γ or de-ADPR-IFN-γ (100 ng/ml). Cells were harvested and solubilized in tissue protein extraction reagent (TPER, Pierce) for 20 min at 4°C. Clarified lysates were size fractionated on precast SDS-PAGE gels (NuPAGE®, Thermo-Fischer) and blotted onto PVDF membranes. Blots were subjected to immunodetection with antibodies directed against β5i and PA28-α (ab3329, Abcam and #2408, Cell Signaling). Immunodetection of β-actin (clone AC-15, Sigma) was performed as a loading control. Bound antibodies were detected with the enhanced chemiluminescent system (GE Healthcare) using peroxidase-conjugated anti-rabbit Ig or anti-mouse Ig antibodies (Jackson Immunoresearch Laboratories).

## Results

### Shed ARTC2.2 Preferentially ADP-Ribosylates IFN-γ

**Figure 1:**
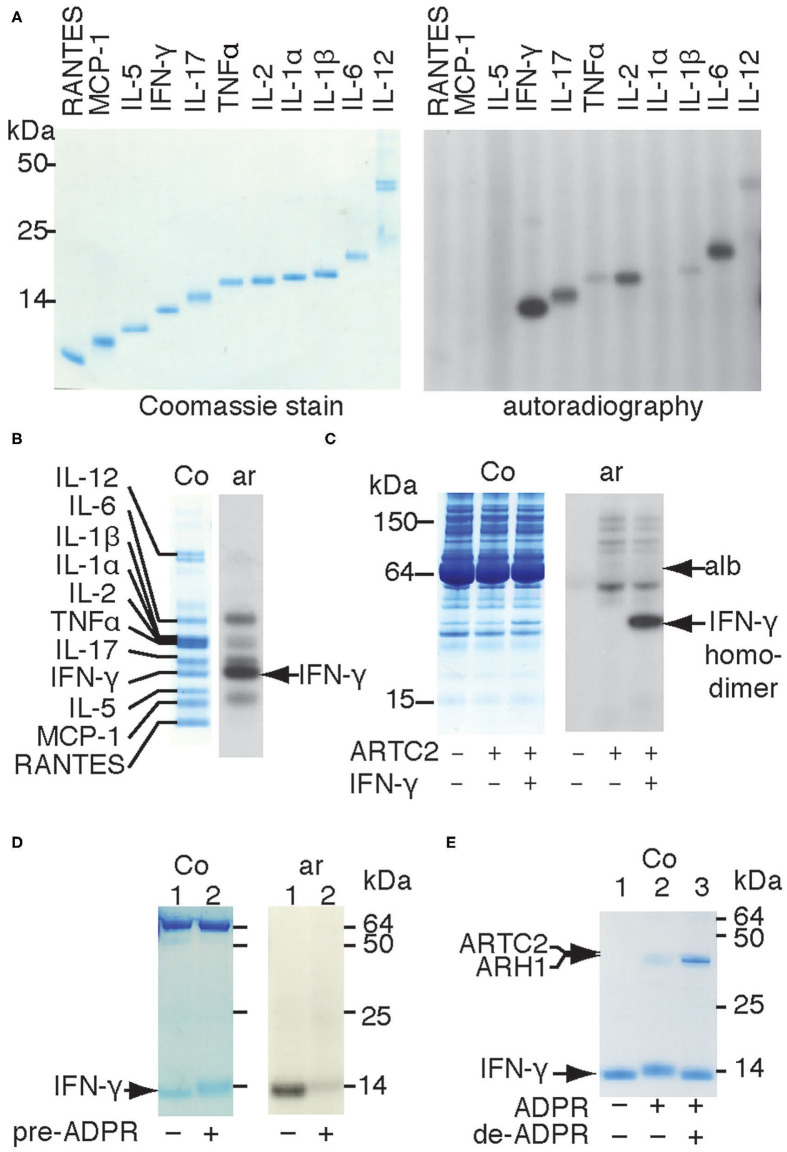
Shed ARTC2.2 preferentially ADP-ribosylates IFN-γ. **(A–D)** Purified shed ARTC2.2 (50 ng) was incubated for 15 min with ^32^P-NAD^+^ (1 μM) and **(A)** individual recombinant cytokines (500 ng), **(B)**, a cocktail of cytokines (500 ng each), **(C)**, serum spiked with IFN-γ, or **(D)** IFN-γ that had been pre-ADP-ribosylated or not by incubation for 30 min with shed ARTC2.2 and non-radioactive NAD^+^. Reactions were stopped by the addition of SDS-PAGE sample buffer. Proteins were size fractionated by SDS-PAGE and stained with Coomassie (Co). ^32^P-ADP-ribosylated proteins were detected by autoradiography (Ar). The gels in A, B, and D were run under reducing conditions, the gel in C was run under non- reducing conditions. In C, alb indicates the prominent 65 kDa band corresponding to endogenous albumin in murine serum. In D the prominent bands at 65 kDa represents bovine albumin that was added as carrier protein. **(E)** IFN-γ was sequentially ADP-ribosylated then de-ADP-ribosylated as described in the methods. Proteins were analyzed by SDS-PAGE and Coomassie-staining. Results are representative of three or four independent experiments.

The observation that shed ARTC2.2 ADP-ribosylates distinct serum proteins (10) prompted us to determine whether ARTC2.2 preferentially ADP-ribosylates certain cytokines. We therefore incubated shed ARTC2.2 with individual recombinant cytokines in the presence of ^32^P-NAD^+^ and monitored covalent incorporation of radioactivity into cytokines by autoradiography after SDS-PAGE (Figure 1 right panel). The results show that shed ARTC2.2 ADP-ribosylates IFN-γ, IL-2, IL-6, and IL-17 (Figure 1 without arrow). When incubated with a cocktail of cytokines, ARTC2.2 potently ADP-ribosylates IFN-γ in addition to other cytokines (Figure 1B, arrow). Similarly, when incubated in serum spiked with IFN-γ, ARTC2.2 preferentially ADP-ribosylated IFN-γ (Figure 1C).

IFN-γ forms a homodimeric bioactive cytokine by a covalent disulfide bridge between the C-terminal cysteine residues (Cys133). Shed ARTC2.2 effectively ADP-ribosylates the native IFN-γ homodimer, as revealed by autoradiography after non-reducing SDS-PAGE (Figure 1C). Analysis of IFN-γ under reducing conditions reveals a discernible shift in migration of the IFN-γ band in Coomassie-stained SDS-PAGE after incubation with shed ARTC2.2 and NAD^+^ (Figures 1D,E, lane 2 vs. lane 1), suggesting that ARTC2.2 ADP-ribosylates both molecules of the IFN-γ homodimer, since the presence of ADP-ribosylated and non-ADP-ribosylated IFN-γ should result in two bands after size fractionation by reducing SDS-PAGE. Consistently, pre-ADP-ribosylation of IFN-γ by preincubation with ARTC2.2 and non-radioactive NAD^+^ for 60 min prevented subsequent incorporation of radiolabel during further incubation with ^32^P-labeled NAD^+^ (Figure 1D). Addition of recombinant ADP-ribosylarginine hydrolase 1 (ARH1) to ADP-ribosylated IFN-γ caused a shift in migration back to that of native IFN-γ (Figure 1E, lane 3), consistent with removal of the ADP-ribose moieties from both molecules of the IFN-γ homodimer. This shift in migration by ADP-ribosylation and de-ADP-ribosylation can be seen in the Coomassie-stained gel without autoradiography (Figure 1E).

**Figure 2:**
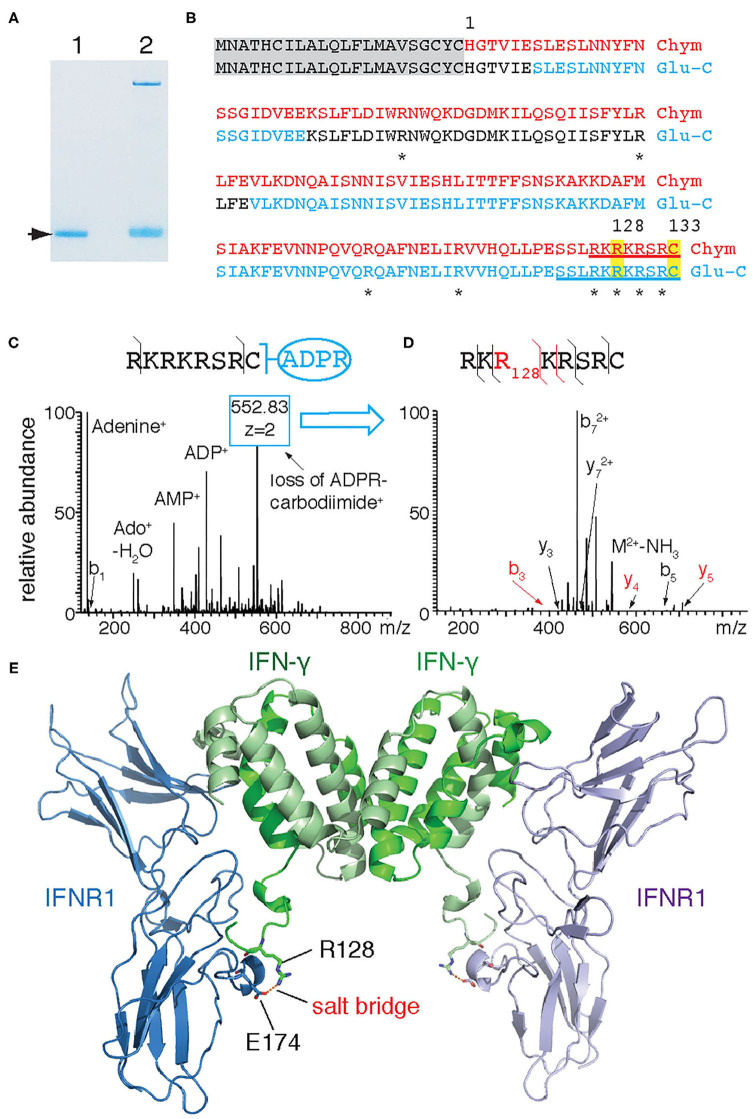
IFN-γ is ADP-ribosylated by ARTC2.2 at R128. **(A)** Recombinant IFN-γ was incubated in the absence (lane 1) or presence (lane 2) of shed ARTC2.2 and NAD^+^. Bands corresponding to unmodified and ADP-ribosylated IFN-γ (arrow) were excised from the gel and subjected to in-gel digestion with chymotrypsin or Glu-C protease. **(B)** Peptides identified by MS-MS analyses are indicated in red and blue for chymotrypsin and Glu-C, respectively. Potential target arginine residues for ADP-ribosylation are marked by asterisks. Peptides exhibiting the characteristic ADP-ribose fragmentation fingerprint are underlined. **(C)** HCD-MS^2^ spectrum of the ADP-ribose-containing C-terminal peptide generated from IFN-γ by chymotrypsin cleavage, showing the characteristic ADP-ribosyl fragments. The most intense peak corresponds to a fragment resulting from the conversion of ADP-ribosyl-arginine to ornithine (loss of ADP-R-carbodiimide). **(D)** MS^3^-CID spectrum of the ornithine-containing peptide generated by MS^2^. **(E)** Model of interaction between murine IFN-γ and IFNR1. IFN-γ and IFNR1 were modeled onto the co-crystal structures of human IFN-γ and *Ectromelia virus* IBP^ECTV^ (PDB ID: 3bes) using Swiss Model. Models were aligned to 3bes in Pymol. R128 of IFN-γ and E174 of IFNR1 are presented as sticks with oxygen atoms in red and nitrogen atoms in blue—the corresponding residues in human IFN-γ and IBP^ECTV^ form a salt bridge. The C-terminal amino acids (R132 and C133) of murine IFN-γ could not be modeled since the corresponding residues of human IFN-γ are not visible in 3bes.

In order to identify the target arginine for ADP-ribosylation of IFN-γ, we subjected ADP-ribosylated recombinant IFN-γ to LC-MS/MS following in-gel digestion with chymotrypsin and Glu-C (Figure 2A). MS^2^ analyses revealed a combined sequence coverage of 100% (Figure 2B red). Both Higher-Energy Collisional Dissociation (HCD) and Collision Induced Dissociation (CID) of ADP-ribose containing peptides yielded a characteristic MS fingerprint with the major sites of fragmentation occurring at the pyrophosphate bond and the terminal adenine. Manual inspection of the HCD-MS^2^ spectra from chymotrypsin-generated peptides revealed a single peptide—R^126^KRKRSRC^133^–exhibiting this characteristic ADP-ribose fingerprint with very little peptide-backbone fragmentation (Figure 2C). This peptide contains four arginines and the protein's C-terminus. Fragmentation of the ADP-ribose moiety during CID results in the conversion of the ADP-ribosylated arginine to ornithine. The intact ornithine containing peptide can be further isolated in tandem mass spectrometry and subjected to MS^3^ analysis for peptide sequencing (17). Applying this strategy to the ADP-ribosylated peptide allowed assignment of ornithine at R128 (Figure 2D). Similarly, LC-MS analyses of peptides generated by in-gel digestion with Glu-C resulted in a single peptide - S^123^SLRKRKRSRC^133^ - exhibiting the characteristic ADP-ribosyl-arginine fragments (Figure 2B). A targeted MS^3^ experiment again assigned R128 as ornithine (not shown). Together, these results unambiguously identify R128 as the target site of IFN-γ for ADP-ribosylation by ARTC2.2. A model of mouse IFN-γ in complex with IFN-γ receptor 1 (IFNR1) based on the published 3D-crystal structure of human IFN-γ with the *Ectromelia virus* IFN-γ binding protein (pdb code 3bes) (18) indicates that R128 participates in binding to IFNR1 by forming a salt bridge to E174 of IFNR1 (Figure 2E).

### ARTC2.2-Catalyzed ADP-Ribosylation Inhibits the Signaling Function of IFN-γ

**Figure 3:**
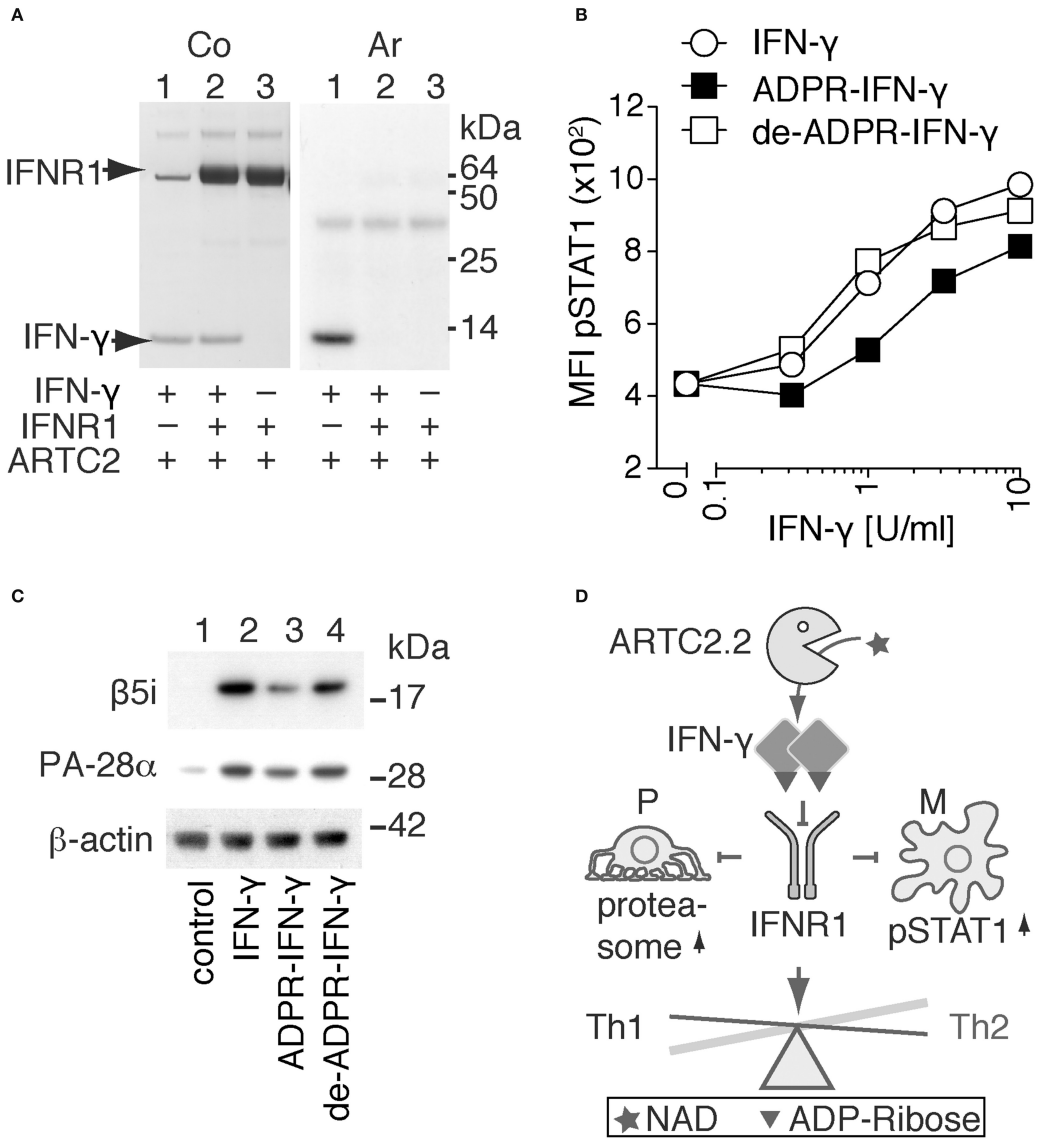
ADP-ribosylation inhibits the signaling function of IFN-γ. **(A)** Recombinant IFN-γ and the ectodomain of IFNR1 were preincubated for 1h at 4°C before addition of shed ARTC2.2 and ^32^P-NAD^+^. After further incubation for 15 min, reactions were stopped by addition of SDS-PAGE sample buffer. Proteins were size-fractionated by SDS-PAGE and visualized by Coomassie staining (Co). ADP-ribosylated proteins were detected by autoradiography (Ar). **(B, C)** IFN-γ signaling assays were performed using unmodified IFN-γ, ADP-ribosylated IFN-γ and de-ADP-ribosylated IFN-γ prepared as described in the methods. **(B)** Peritoneal macrophages were incubated for 20 min with serial titrations of IFN-γ, ADP-ribosylated IFN-γ (ADPR-IFN-γ), or de-ADP-ribosylated IFN-γ (de-ADPR- IFN-γ). Cells were then fixed, permeabilized, and stained with a fluorochrome-conjugated mAb against phosphorylated STAT1 (pSTAT1) before flow cytometry analyses. **(C)** Differentiated podocytes were incubated for 24 h in the absence of IFN-γ (control), or presence of either IFN-γ, ADPR-IFN-γ, or de-ADPR-IFN-γ. Cells were washed and lysed in TPER. Insoluble material was removed by centrifugation and solubilized proteins were analyzed by SDS-PAGE and immunoblot analyses. Components β5i of the immunoproteasome and the proteasomal activator PA28-α and β-actin as loading control were detected with appropriate primary and secondary antibodies. Results are representative of three independent experiments. **(D)** Schematic model illustrating the proposed regulation of IFN-γ signaling by ADP-ribosylation. ARTC2.2-catalyzed ADP-ribosylation of IFN-γ inhibits IFNR1 activation and thereby phosphorylation of STAT1 and activation of the immunoproteasome in macrophages (M) and podocytes (P), respectively. By inhibiting IFN-γ signaling, this pathway may tip the balance from a Th1 toward a Th2 response.

On the basis of the structure model of IFN-γ in complex with IFNR1 (Figure 2E), we hypothesized that R128 is not accessible for ADP-ribosylation when IFN-γ is bound to IFNR1. To test this hypothesis, we allowed IFN-γ to form a complex with the recombinant extracellular domain of IFNR1 and analyzed whether IFN-γ is still accessible in this complex for ADP-ribosylation by shed ARTC2.2. The results show that ADP-ribosylation of IFN-γ is blocked in the presence of IFNR1 and that IFNR1 itself is not a target for ADP-ribosylation (Figure 3A).

In order to determine whether ADP-ribosylation of IFN-γ impinges on its signaling function, we used two established cellular assays, that is, IFN-γ-induced phosphorylation of signal-transducer-and-activator-of-transcription-1 (STAT1) in macrophages and IFN-γ-induced expression of the immune proteasome component β5i and the proteasomal activator PA28-α in podocytes (Figures 3B,C). The results show that ADP-ribosylation reduces potency of IFN-γ to induce phosphorylation of STAT1 in primary peritoneal macrophages by approximately four-fold (Figure 3B). De-ADP-ribosylation of IFN-γ with ARH1 restores its original potency to induce STAT1 phosphorylation. Similarly, ADP-ribosylation of IFN-γ inhibits its capacity to induce expression of β5i and PA28-α by murine podocytes (Figure 3C, lane 3), whereas de-ADP-ribosylation of IFN-γ by ARH1 restores its capacity to induce expression of β5i and PA28-α (Figure 3C, lane 4).

## Discussion

Our results confirm that shed ARTC2.2 preferentially ADP-ribosylates secretory proteins (10). To identify possible soluble targets we performed a comparative radio-ADP-ribosylation assay with different cytokines and recombinant soluble ARTC2.2 and ^32^P-NAD^+^. In these experiments ARTC2.2 showed a propensity to ADP-ribosylate IFN-γ. Moreover, we identified R128 as the ADP-ribosylation site of IFN-γ and showed that ADP-ribosylation of IFN-γ inhibits its signaling function.

Arginine ADP-ribosylation is a reversible posttranslational protein modification: ARH1 catalyzes hydrolysis of the ADP-ribose residue from arginine (19). Our results show that recombinant ARH1 can de-ADP-ribosylate IFN-γ and thereby restore the signaling capacity of IFN-γ. In this context, it is important to note that ARH1 lacks an N-terminal signal sequence for export from cells by a classical secretion pathway via the ER and Golgi apparatus and, thus, presumably is expressed as a cytosolic enzyme. It remains to be established whether ARH1 can be released from cells by non-classical secretion system, for example, analogous to other cytosolic enzymes and cytokines that lack a signal sequence such as adenosine deaminase, PBEF1/NAMPT, IL-1β and IL-18 (20–22).

Using LC-MS/MS, we identified R128 as the target site for ADP-ribosylation by ARTC2.2 (Figure 2). This site was confirmed in peptides derived from two different proteases (chymotrypsin, Glu-C). R128 is located in a highly basic region, just five amino acid residues upstream of the C-terminal cysteine. Arginines in other proteins that function as ADP-ribosylation targets of vertebrate ARTCs typically lie in regions with high pKa (23). Consistently, R128 is flanked on either side by lysine and arginine residues. Previous functional and structural studies revealed a crucial role of the C-terminus of IFN-γ for receptor binding and biological activity (18, 24–26). Deletion of the C-terminus or site directed mutagenesis of the basic residues in this region markedly reduce the capacity of IFN-γ to bind to its receptor (24, 26). Importantly, site-directed mutagenesis of R129 of human IFN-γ–that is, the residue corresponding to the R128 ADP-ribosylation site of murine IFN-γ–drastically reduced receptor binding and biological activity (25).

The C-terminus is not resolved in known structures of IFN-γ, either alone or in complex with its receptor, IFNR1 (27, 28). However, R129 of human IFN-γ is visible in the co-crystal structure of IFN-γ bound to IBP^ECTV^, a viral protein homologous to IFNR1. Interestingly, this residue forms a salt bridge to a conserved aspartic acidic residue in IBP^ECTV^ (18) that corresponds to E174 of IFNR1. It is thus conceivable that a bulky, negatively charged ADP-ribose moiety at R128 would disturb this interaction and lead to reduced biological activity.

IFN-γ is secreted mainly by activated Th1 effector cells and is an important endogenous mediator of immunity and inflammation. It plays a key role in macrophage activation and T cell differentiation (29). Excessive IFN-γ can lead to tissue damage and autoimmunity (26, 30, 31) as illustrated by the severe inflammatory disease in mice deficient in suppressor of cytokine signaling 1 (SOCS1), one of several known attenuators of IFN-γ expression (32). It was also shown that C-terminal truncation of IFN-γ by matrix metalloproteinase 12 controls IFN-γ signaling to resolve inflammation and that this negative feedback mechanism is often defective in autoimmune diseases (26).

Recent studies indicate that the IFN-γ/STAT-1 signaling pathway in macrophages regulates and is regulated also by intracellular ADP-ribosylransferases, for example, ARTD8 and ARTD9 (33–37). Interestingly IFN-γ signaling stimulates the activity of these members of the ARTD family, and these enzymes in turn modulate IFN-γ signaling by ADP-ribosylating STAT1.

ADP-ribosylation provides an additional, posttranslational means for regulating IFN-γ signaling. However, given the locally restricted release of ARTC2.2 and IFN-γ from activated T cells, and of NAD^+^ as substrate from lysed or damaged cells, the total amount of ADP-ribosylated IFN-γ *in vivo* is likely to be low. Currently available tools lack the sensitivity to monitor ADP-ribosylation of IFN-γ *in vivo*. ARTC2.2 is expressed in mice and other rodents but is not expressed in humans and other primates which carry a nonfunctional ARTC2.2. pseudogene (11). Since ADP-ribosylation of IFN-γ is mediated by soluble ARTC2.2, it is conceivable that other members of the ADP-ribosytransferase family for example, ARTC1 (7, 12) could replace this function in humans.

Considering that IFN-γ drives differentiation of effector T cells to Th1 cells, and conversely that neutralizing IFN-γ promotes differentiation to Th2 cells, we hypothesize that ADP-ribosylation of IFN-γ might be involved in endogenous regulatory mechanisms to tip the balance from a Th1 to a Th2 immune response and thereby to diminish unwanted autoimmune responses (Figure 3D).

In summary, our results uncover a hitherto unrecognized mechanism to control signaling by IFN-γ *via* reversible ADP-ribosylation by the shed ectodomain of ARTC2.2. Our findings point to new avenues for modulating inflammatory reactions by pharmacologically blocking or enhancing ADP-ribosylation of IFN-γ, for example, by suitable decoy-peptides or by targeting recombinant ecto-ADP-ribosyltransferase ARTC2.2 or ADP-ribosylarginine hydrolase ARH1 to IFN-γ at sites of inflammation.

## Data Availability Statement

The raw data supporting the conclusions of this article will be made available by the authors, without undue reservation.

## Ethics Statement

The animal study was reviewed and approved by Hamburger Behörde für Gesundheit und Verbraucherschutz, Veterinärwesen/Lebensmittelsicherheit, ORG 153.

## Author Contributions

SM, FH, CM-S, AT, and FK-N conceived the project. SM and FK-N wrote the manuscript. SM performed the experiments shown in Figures 1, 2A,E and 3A. TK those in Figures 2B–D. BR those in Figure 3B. CM-S those in Figure 3C. All authors reviewed and approved the manuscript.

## Conflict of Interest

The authors declare that the research was conducted in the absence of any commercial or financial relationships that could be construed as a potential conflict of interest.

## Standard Abbreviations

Ab, ATP, LPS, C-terminal, N-terminal, Da, EC_50_, FCS, i.v. IFN, IL, GPI, NAD, mAb.

## Abbreviations

ARTC: ADP-ribosyltransferase subgroup related to Clostridial C2/C3 toxins
ARTD: ADP-ribosyltransferase subgroup related to Diphtheria toxin
ARH: arginine ADP-ribosylhydrolase.
